# Efficacy of stem cell therapy for burn wounds: a systematic review and meta-analysis of preclinical studies

**DOI:** 10.1186/s13287-020-01839-9

**Published:** 2020-07-29

**Authors:** Yuan Li, Wei-dong Xia, Leanne Van der Merwe, Wen-tong Dai, Cai Lin

**Affiliations:** 1 Department of Burn, the First Affiliated Hospital of Wenzhou Medical University, Nan Bai Xiang, Wenzhou, Zhejiang, 325000 People’s Republic of China; 2 School of International Studies, Wenzhou Medical University, Wenzhou, Zhejiang, 325000 People’s Republic of China

**Keywords:** Burns, Stem cell therapy, Wound healing, Meta-analysis, Preclinical study

## Abstract

**Background:**

Burns remain a serious public health problem with high morbidity and mortality rates worldwide. Although there are various treatment options available, there is no consensus on the best treatment for severe burns as of yet. Stem cell therapy has a bright prospect in many preclinical studies of burn wounds. The systematic review was performed for these preclinical studies to assess the efficacy and possible mechanisms of stem cells in treating burn wounds.

**Methods:**

Twenty-two studies with 595 animals were identified by searching PubMed, EMBASE, Web of Science, and Cochrane Library databases from inception to 13 May 2020. In addition, a manual search of references of studies was performed to obtain potential studies. No language or time restrictions were enforced. RevMan 5.3 was used for all data analysis.

**Results:**

The overall meta-analysis showed that stem cell therapy significantly improved burn healing rate (SMD 3.06, 95% CI 1.98 to 4.14), irrespective of transplant type, burn area, and treatment method in the control group. Subgroup analyses indicated that hair follicle stem cells seemed to exert more beneficial effects on animals with burn wounds (SMD 7.53, 95% CI 3.11 to 11.95) compared with other stem cells. Furthermore, stem cell therapy seemed to exert more beneficial effects on burn wounds with second-degree (SMD 7.53, 95% CI 3.11 to 11.95) compared with third-degree (SMD 2.65, 95% CI 1.31 to 4.00).

**Conclusions:**

Meta-analysis showed that stem cell therapy exerts a healing function for burn wounds, mainly through angiogenesis and anti-inflammatory actions. These findings also demonstrate the need for considering variations in future clinical studies using stem cells to treat a burn wound in order to maximize the effectiveness. In general, stem cells can potentially become a novel therapy candidate for burn wounds.

## Introduction

Even at current medical levels, burn remains a serious public health problem with high morbidity and mortality worldwide [1, 2]. The World Health Organization indicated that nearly 300,000 deaths occur annually, worldwide, from burns, but most of them not caused by fatal burns [3]. After effective and timely treatment, many patients can retain a considerable quality of life. The primary goal of burn treatment is effective wound management, which largely determines the survival and prognosis of patients with severe burns [4, 5]. Although the skin has the ability to heal itself, severe burns require a variety of interventions, such as healing drugs [6, 7], debridement [8, 9], and skin grafts [10, 11]. However, for severe burns, skin grafts can cause harmful psychological effects [12] and severe disfigurement of the donor’s skin [13]. Subsequently, the formation of scar and contracture will lead to considerable decrease of joint activity, and even the loss of function [14]. Various healing drugs including DNA [15], stem cells [16, 17], growth factors [18, 19], and siRNA [20] have been pursued to promote burn wound repair and regeneration. Although there are various treatment options, there is no consensus yet on the best treatment for severe burns such as deep partial-thickness and full-thickness burns. Therefore, more effective burn treatment drugs are urgently needed to treat burn wounds.

Stem cell therapy is an emerging method based on proliferation and/or differentiation of transplanted stem cells to repair or even replace damaged tissues or organs, which in effect offers new possibilities for regenerative medicine [21, 22]. Furthermore, stem cells are abundant in origin and can be isolated from adipose tissue, umbilical cord, embryo, bone, gingiva, and other tissues [23]. It is reported that stem cell transplantation has been applied to treat various disease models and significantly improved their prognosis, including burns [24], digestive diseases [25], renal diseases [26], and autoimmune diseases [27]. In recent years, stem cell therapy has attracted increasing interest as a potential treatment for burn wounds, because stem cells may affect many processes of burn wound healing, including accelerating the synthesis of the extracellular matrix (ECM), alleviating the inflammatory response, and promoting the angiogenesis [16, 28–30]. Although clinical trials have been reported [31, 32], most of the studies on stem cell-mediated repair of burn wounds have been conducted in animal models. Animal experiment has its special approach in increasing the understanding of the physiological and pathological processes of a disease, which lays a foundation for future clinical trials. In addition, preclinical reviews can more systematically evaluate the mechanisms of stem cell efficacy and provide vital evidence for stem cell research. Thus, we aimed to perform a systematic review for these preclinical studies to assess the efficacy and possible mechanisms of stem cells in treating burn wounds.

## Methods

This review adheres to the guidelines outlined in the Preferred Reporting Items for Systematic Reviews and Meta-Analyses guidelines [33]. Supplementary Table 1 shows the PRISMA 2009 checklist. The detailed protocol is registered through PROSPERO (CRD42020186182), which can be found online at https://www.crd.york.ac.uk/prospero/display_record.php?ID=CRD42020186182.

### Literature search

We conducted a thorough search to assess the association between stem cell therapy and burns. PubMed, EMBASE, Cochrane Library, and Web of Science were searched from their inception to 13 May 2020. The search phrases used in PubMed are as follows: “epidermal stem cells,” “mesenchymal stromal cells,” “mesenchymal stem cell,” “adipose-derived stem cells,” “stem cells,” or “stem cell” paired with “burns” or “burn”. The search was limited to animal trial studies and be published English. In addition, we performed a manual search of references of studies to obtain potential studies.

### Study selection

Inclusion criteria for studies were prespecified as follows: (1) reported as a randomized controlled trial (RCT); (2) experimental animal models of burns; (3) experimental group received stem cell therapy (mesenchymal stem cells, adipose-derived stem cells, etc.); (4) control group received only nonfunctional solutions, vehicle, or no treatment; and (5) the primary outcome was the healing rate of burns. The secondary outcomes were collagen deposition, blood vessel density, and inflammatory markers. Exclusion criteria for studies were prespecified as follows: (1) no control group in the study or comparing stem cell with another therapy; (2) case report, review, and clinical trial; (3) lack of available data; and (4) repeated publication.

### Data extraction

Two authors independently extracted detailed information from the included studies, and the disagreement was resolved by a third author. The following data were collected: (1) the first author and publication year; (2) countries of the studies; (3) animal characteristics (including species, number, burn degree, and area); (4) administration methods of treatment group and control group; (5) stem cell information (including cell type, number, origin, and transplant type); and (6) primary and secondary outcomes.

If the results continuously increase or decrease at multiple time points, only the last time point will be selected for analysis. If the result fluctuates during the treatment, only the highest or lowest value at the first increase or decrease will be selected. When the data results were only presented in the form of pictures, we tried to obtain the data by contacting the author. If a poor response from the author, digital ruler software was used in order to measure numerical values.

### Quality assessment

The risk of bias was assessed by two independent reviewers using a ten-item scale [34] for animal studies, with minor modifications. Aspects of risk of bias include sequence generation, baseline characteristics, allocation concealment, random housing, blinding of investigators, random animals assessment, blinding of outcome assessor, incomplete outcome data, selective outcome reporting, and other sources of bias.

### Statistical analysis

All statistical analysis was performed with RevMan V.5.3 software. All outcomes were regarded as continuous data and presented as standard mean difference (SMD) with 95% CIs (confidence intervals). The Cochrane *Q*-statistic test and the *I*^2^-statistic test were applied to evaluate heterogeneity among the studies, and a *P* < 0.05 was regarded statistically effective. An *I*^2^ of higher than 50% was considered an indicator of statistically significant heterogeneity among the studies [35]. If the study was homogeneous, a fixed-effects model was adopted; if there was heterogeneity between studies, a random-effects model was used. Subgroup analysis or sensitivity analysis was performed when inter-study heterogeneity existed. Funnel plots were used to assess the publication bias when there were more than nine studies in that outcome.

## Results

### Study selection

**Fig. 1:**
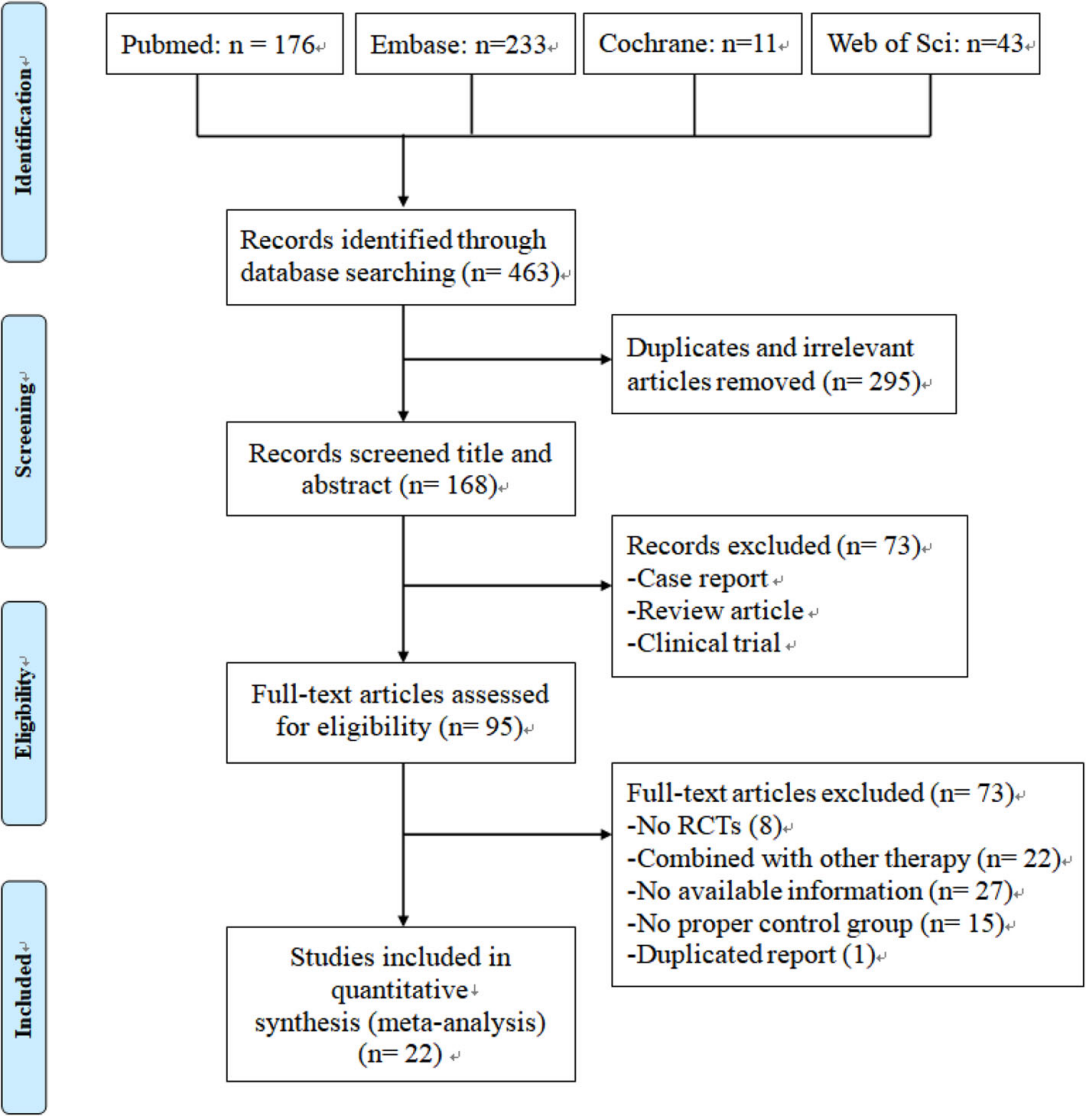
Flowchart of the details of study selection

In total, 463 records were identified in the initial search of the four databases, and 295 were removed mainly because they were duplicates or irrelevant to our objective. After title and abstract screening, 73 studies were removed for various reasons such as reviews, clinical experiments, and case reports. After careful full-text of the remaining 95 articles, 73 were excluded for the following reasons: (1) failed to obtain available information, (2) stem cell therapy combined with other therapy in experimental group, (3) no proper control group, (4) duplicated report of the same study, and (5) not randomized controlled trials. Ultimately, 22 studies [16, 17, 28–30, 36–52] were included in our systematic review and meta-analysis (Fig. 1).

### Characteristics of eligible studies

The general characteristics of the 22 articles are summarized in Table 1. All studies were published in English between 2013 and 2020. Fourteen studies [16, 28, 29, 37–39, 41, 44–50] used rats, six studies [17, 30, 40, 42, 43, 51] used mice, and two studies [36, 52] used minipigs. A total of 10 studies were conducted in China [29, 30, 37, 39, 40, 43, 47–49, 51], 3 studies in Brazil [16, 28, 41], 3 studies in the USA [36, 42, 52], and 2 studies in Iran [38, 45], whereas the remaining 4 were conducted in Pakistan [46], Egypt [44], Turkey [50], and Canada [17], respectively. Four studies [40, 43, 48, 51] did not report burn degree and two [38, 45] used second-degree burns, while the others used third-degree. The cell types used for transplantation were mesenchymal stem cells (MSCs) [17, 28–30, 43–48, 50], epidermal stem cells (ESCs) [40, 51], hair follicle stem cells (HFSCs) [38], stromal vascular fraction (SVF) [36, 52], and adipose-derived stem cells (ASCs) [16, 37, 39, 41, 42, 49]. As for mesenchymal stem cells, they derived from umbilical cord [30, 46–48], bone marrow [28, 29, 43–45, 50], and burned tissue [17]. The types of transplant for cell therapy include xenogenic [28, 30, 41–43, 45–48], allogeneic [16, 29, 37–40, 44, 50, 51], and autologous [17, 36, 39, 49, 52]. It is worth noting that one study [39] reported the use of allogeneic stem cells and xenogenic stem cells. Except for three studies [30, 45, 46] that did not report the dose of stem cells, the dose of stem cells in the remaining studies was 0.5–21 × 10^6^. Except for two studies [30, 45] that did not report the intervention method and one study that treated by intravenous injection [52], the interventions in other studies were treated by subcutaneous injection. It is worth mentioning that two studies [36, 52] used two different methods of stem cell interventions. In terms of placebo, eight studies did not use any treatment in the control group [16, 17, 40, 44–46, 48, 51], 10 studies used PBS [28, 29, 37, 38, 41–43, 47, 49, 50], two studies used medium [30, 39], and two studies used lactate ringer [36, 52]. All studies reported at least one predetermined outcome measure.

**Table 1:** Characteristics of the included studies

Study (year)	Country	Animal (number)	Burn area (cm^2^)	Burn degree	Cell type	Origin	Transplant type	Cell number	Method	Placebo	Outcome index
Abbas et al., 2018 [50]	Turkey	Wistar rats (40)	10.45	Third	MSCs	Bone marrow	Allogenic	1 × 10^6^	Subcutaneous injection	PBS	(1) Blood vessel density, (2) IL-1, (3) VEGF, (4) TNF-α
Amini-Nik et al., 2018 [17]	Canada	Nude mice (10)	25	Third	MSCs	Burned tissue	Autologous	5 × 10^6^	Subcutaneous injection	No	(1) Wound healing rate
de Andrade et al., 2020 [41]	Brazil	Wistar rats (48)	20	Third	ASCs	Adipose	Xenogenic	1.5 × 10^6^	Subcutaneous injection	PBS	(1) Wound healing rate, (2) collagen I and III, (3) VEGF
Aryan et al., 2018 [45]	Iran	Wistar rats (32)	9	Second	MSCs	Bone marrow	Xenogenic	NA	NA	No	(1) Blood vessel density
Babakhani et al., 2020 [42]	Iran	Wistar rats (30)	2.25	Second	HFSCs	Hair follicle	Allogenic	1 × 10^6^	Subcutaneous injection	PBS	(1) Wound healing rate, (2) total collagen
Bliley et al., 2016 [38]	USA	Nude mice (24)	0.79	Third	ASCs	Adipose	Xenogenic	6.8 × 10^6^	Subcutaneous injection	PBS	(1) Blood vessel density, (2) collagen I and III
Caliari-Oliveira et al., 2016 [28]	Brazil	Wistar rats (28)	45	Third	MSCs	Bone marrow	Xenogeneic	5 × 10^6^	Subcutaneous injection	PBS	(1) Wound healing rate, (2) blood vessel density, (3) total collagen
Chang et al. #1, 2018 [39]	China	Wistar rats (6)	0.5	Third	ASCs	Adipose	Autologous	5 × 10^6^	Subcutaneous injection	Medium	(1) Wound healing rate
Chang et al. #2, 2018 [39]	China	Wistar rats (6)	0.5	Third	ASCs	Adipose	Allogenic	5 × 10^6^	Subcutaneous injection	Medium	(1) Wound healing rate
Feng et al., 2019 [37]	China	SD rats (18)	1	Third	ASCs	Adipose	Allogenic	5 × 10^5^	Subcutaneous injection	PBS	(1) Blood vessel density
Foubert et al. #1, 2016 [36]	USA	Gottingen minipigs (10)	10	Third	SVF	Adipose	Autologous	2.5 × 10^6^	Subcutaneous injection	LR	(1) Wound healing rate, (2) blood vessel density
Foubert et al. #2, 2016 [36]	USA	Gottingen minipigs (10)	10	Third	SVF	Adipose	Autologous	2.5 × 10^6^	Spray	LR	(1) Wound healing rate, (2) blood vessel density
Foubert et al. #1, 2017 [52]	USA	Gottingen minipigs (8)	10	Third	SVF	Adipose	Autologous	19.5 × 10^6^	Subcutaneous injection	LR	(1) Blood vessel density
Foubert et al. #2, 2017 [52]	USA	Gottingen minipigs (10)	10	Third	SVF	Adipose	Autologous	21 × 10^6^	Intravenous injection	LR	(1) Blood vessel density
Franck et al., 2019 [16]	Brazil	Wistar rats (23)	4.84	Third	ASCs	Adipose	Allogenic	3.2 × 10^6^	Subcutaneous injection	No	(1) Total collagen, (2) collagen I and III
Imam et al., 2019 [44]	Egypt	Albino rats (20)	2	Third	MSCs	Bone marrow	Allogenic	1 × 10^6^	Subcutaneous injection	No	(1) IL-1, (2) VEGF
Li et al., 2019 [29]	China	Wistar rats (18)	NA	Third	MSCs	Bone marrow	Allogenic	1 × 10^6^	Subcutaneous injection	PBS	(1) IL-1, (2) VEGF, (3) TNF-α
Liu et al., 2014 [47]	China	Wistar rats (28)	NA	Third	MSCs	Umbilical cord	Xenogenic	5 × 10^6^	Subcutaneous injection	PBS	(1) Wound healing rate, (2) blood vessel density, (3) IL-1, (4) VEGF, (5) TNF-α
Mahmood et al., 2019 [46]	Pakistan	SD rats (12)	4	Third	MSCs	Umbilical cord	Xenogeneic	NA	Subcutaneous injection	No	(1) Wound healing rate, (2) VEGF
Xue et al., 2013 [43]	China	Mice (20)	NA	NA	MSCs	Bone marrow	Xenogenic	1 × 10^6^	Subcutaneous injection	PBS	(1) Wound healing rate, (2) blood vessel density
Yang et al., 2019—1 [51]	China	BALB/c mice (30)	1	NA	ESCs	Epidermis	Allogenic	3.2 × 10^6^	Subcutaneous injection	No	(1) Wound healing rate, (2) blood vessel density,
Yang et al., 2019—2 [40]	China	BALB/c mice (30)	1	NA	ESCs	Epidermis	Allogenic	3.2 × 10^6^	Subcutaneous injection	No	(1) Wound healing rate, (2) blood vessel density
Zhang et al., 2015 [48]	China	SD rats (84)	1.77	NA	MSCs	Umbilical cord	Xenogeneic	2 × 10^6^	Subcutaneous injection	No	(1) Wound healing rate, (2) TNF-α
Zhou et al., 2019—1 [30]	China	C57BL/6 mice (36)	2.25	Third	MSCs	Umbilical cord	Xenogenic	NA	NA	Medium	(1) Wound healing rate, (2) blood vessel density
Zhou et al., 2019—2 [49]	China	SD rats (18)	2	Third	ASCs	Adipose	Autologous	6 × 10^6^	Subcutaneous injection	PBS	(1) Blood vessel density

*ASCs* adipose-derived stem cells, *ESCs* epidermal stem cells, *HFSCs* hair follicle stem cells, *IL-1* Interleukin-1, *LR* lactate ringer, *MSCs* mesenchymal stem cells, *NA* not available, *PBS* phosphate-buffered saline, *SD* Sprague Dawley, *SVF* stromal vascular fraction, *TNF-α* tumor necrosis factor-α, *VEGF* vascular endothelial growth factor

### Quality assessment

The overall quality scores of the included studies ranged from 3 to 6, as shown in Table 2. In all 22 included studies, 41% (*n* = 9) [17, 29, 30, 38, 41, 45, 47, 48, 50] were considered low risk of bias under randomization to burn model or grouping. While all included studies reported the baseline characteristics, the risk of bias was unclear as to allocation concealment. Only two studies [40, 50] have reported using random housing in experimental designs. As for blinding, blinding of investigators was used in only 2 studies [40, 51] and blinding of outcome assessor was used in 10 studies [28, 30, 36, 38, 40, 41, 44, 47, 51, 52]. Six studies [30, 45–47, 49, 50] described that animals were selected at random for outcome assessment. All included studies are considered to have no selective outcome reporting and complete reporting of all outcomes, while other sources of risks are unclear.

**Table 2:** Risk of bias of the included studies

Study	A	B	C	D	E	F	G	H	I	J	Total
Abbas et al. [50]	+	+	–	+	?	+	?	+	+	?	6
Amini-Nik et al. [17]	+	+	–	–	?	?	?	+	+	?	4
de Andrade et al. [41]	+	+	–	–	?	?	+	+	+	?	5
Aryan et al. [45]	+	+	–	–	?	+	?	+	+	?	5
Bliley et al. [38]	+	+	–	–	?	?	+	+	+	?	5
Babakhani et al. [42]	?	+	–	–	?	?	?	+	+	?	3
Caliari-Oliveira et al. [28]	?	+	–	–	?	?	+	+	+	?	4
Chang et al. [39]	?	+	–	–	?	?	?	+	+	?	3
Feng et al. [37]	?	+	–	–	?	?	?	+	+	?	3
Foubert et al. [36]	?	+	–	–	?	?	+	+	+	?	4
Foubert et al. [52]	?	+	–	–	?	?	+	+	+	?	4
Franck et al. [16]	?	+	–	–	?	?	?	+	+	?	3
Imam et al. [44]	?	+	–	–	?	?	+	+	+	?	4
Li et al. [29]	+	+	–	–	?	?	?	+	+	?	4
Liu et al. [47]	+	+	–	–	?	+	+	+	+	?	6
Mahmood et al. [46]	?	+	–	–	?	+	?	+	+	?	4
Xue et al. [43]	?	+	–	–	?	?	?	+	+	?	3
Yang et al.—1 [51]	?	+	–	–	+	?	+	+	+	?	5
Yang et al.—2 [40]	?	+	–	+	+	?	+	+	+	?	6
Zhang et al. [48]	+	+	–	–	?	?	?	+	+	?	4
Zhou et al.—1 [30]	+	+	–	–	?	+	+	+	+	?	6
Zhou et al.—2 [49]	?	+	–	–	?	+	?	+	+	?	4

Note: Studies fulfilling the criteria of the following: *A*, sequence generation; *B*, baseline characteristics; *C*, allocation concealment; *D*, random housing; *E*, blinding of investigators; *F*, random animals assessment; *G*, blinding of outcome assessor; *H*, incomplete outcome data; *I*, selective outcome reporting; and *J*, other sources of bias

### Primary outcome

#### Burn healing rate

**Fig. 2:**
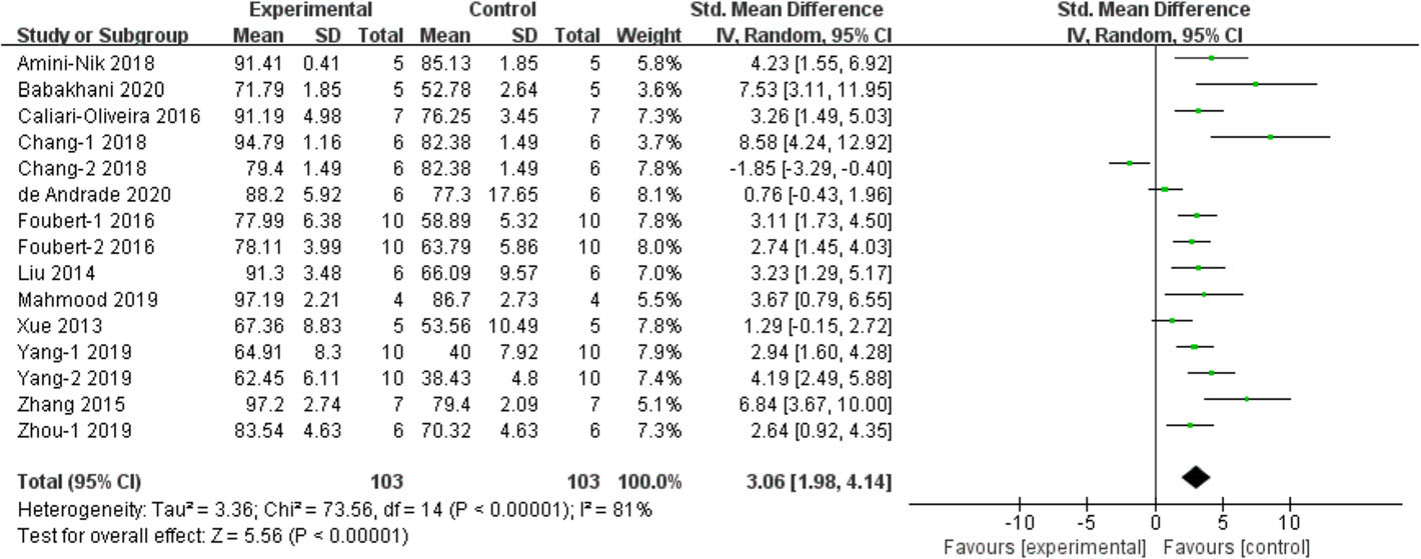
The forest plot: the effects of stem cell therapy for increasing healing rate of burn wounds compared with controls

Meta-analysis of 13 studies [17, 28, 30, 36, 38–41, 43, 46–48, 51] showed that stem cells induces a significant promotion in healing rate of burn animals, compared with control (*n* = 206 SMD 3.06, 95% CI (1.98 to 4.14), *P* < 0.00001; *χ*^2^ = 73.56, *I*^2^ = 81%) (Fig. 2).

Sensitivity analysis was performed because of the high statistical heterogeneity of the meta-analysis. However, the heterogeneity remained after excluding each of these studies in turn (data not shown). In addition, subgroup analysis was grouped according to the following themes: stem cell type, transplant type, burn degree, burn area, treatment method in control group, and species. Notably, the cell type of HFSC demonstrated more efficacy in promoting wound healing, compared to other cell types (SMD 7.53, 95% CI 3.91 to 11.95; Supplementary Fig. 1). Burn wound treatment with MSCs (SMD 3.22, 95% CI 2.09 to 4.36), SVF (SMD 3.06, 95% CI 1.98 to 4.14), and ESCs (SMD 3.45, 95% CI 2.25 to 4.65) also showed significant efficacy compared with ASCs (SMD 1.75, 95% CI − 1.82 to 5.31). By comparing burn wound healing rate from different transplant types, we discovered that autologous stem cells (SMD 3.74, 95% CI 2.21 to 5.27) did not provide a significantly better therapeutic effect than either allogeneic (SMD 2.85, 95% CI − 0.50 to 6.20) or xenogenic stem cells (SMD 2.73, 95% CI 1.49 to 3.97, Supplementary Fig. 2). When comparing the studies in different burn degrees, stem cell therapy on second-degree burn wounds showed a more significant effect compared with third-degree burn wounds (SMD 7.53 vs 2.65; Supplementary Fig. 3). Nonetheless, this result might be subjected to other factors. For example, only one study using a second-degree burn model reported the results of wound healing. It is worth noting that stem cell therapy seemed to exert similar beneficial effects on animals with burn area < 5 cm^2^ (SMD 3.91, 95% CI 1.70 to 6.11; *P* < 0.00001) and ≥ 5 cm^2^ (SMD 2.62, 95% CI 1.46 to 3.78; *P* < 0.00001) (Supplementary Fig. 4). Five studies compared stem cells treatment with phosphate-buffered saline (PBS), three studies compared stem cells treatment with medium, two studies compared stem cell treatment with lactate ringer, and five studies compared stem cell treatment with no treatment. There was no significant difference in the results of different treatment methods in the control group (Supplementary Fig. 5). By comparing different animal species treated with stem cells, we discovered that stem cell therapy has been shown to be effective in mice (SMD 2.89, 95% CI 1.82 to 3.96), rats (SMD 3.54, 95% CI 1.33 to 5.75), and minipigs (SMD 2.91, 95% CI 1.97 to 3.86, Supplementary Fig. 6).

### Secondary outcomes

#### Blood vessel density

**Fig. 3:**
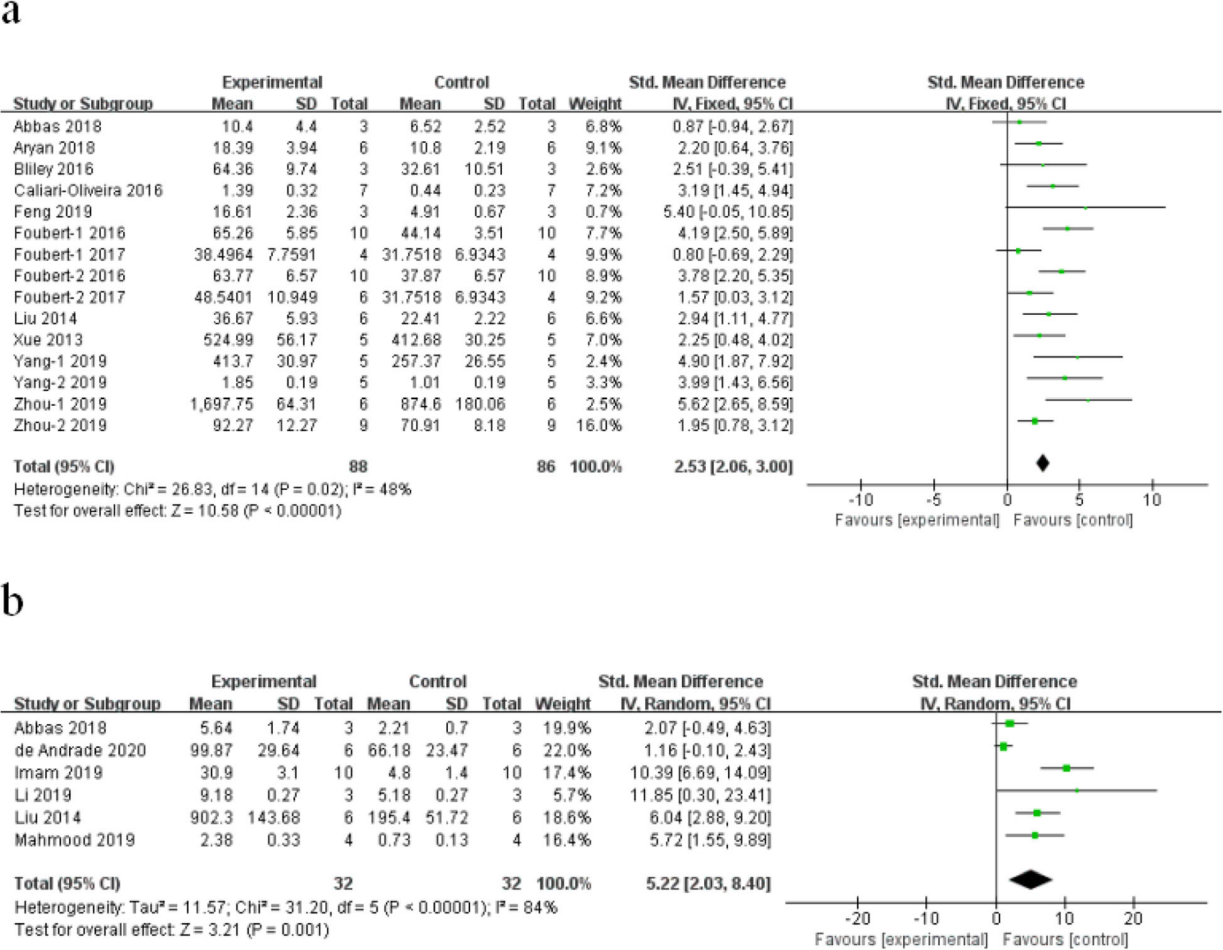
The forest plot: the effects of stem cell therapy for **a** increasing blood vessel number and **b** increasing the level of VEGF on burn wounds compared with controls

Meta-analysis of 13 studies [28, 30, 36, 37, 40, 42, 43, 45, 47, 49–52] showed that stem cells induces a significant promotion in angiogenesis of burn wounds, compared with control (*n* = 174 SMD 2.53, 95% CI (2.06 to 3.00), *P* < 0.00001; *χ*^2^ = 26.83, *I*^2^ = 48%) (Fig. 3a). Meta-analysis of six studies [29, 41, 44, 46, 47, 50] showed that stem cells induce a significant upregulation in the expression of vascular endothelial growth factor (VEGF) in burn wounds, compared with control (*n* = 64 SMD 5.22, 95% CI (2.03 to 8.40), *P* = 0.001; *χ*^2^ = 31.20, *I*^2^ = 84%) (Fig. 3b).

#### Collagen deposition

Meta-analysis of three studies [16, 28, 38] showed that there was no statistical difference in total collagen deposition at wound site between treatment group and control group (*P* = 0.07) (Supplementary Fig. 7a). Meta-analysis of three studies [16, 41, 42] showed that there was no statistical difference in collagen I and III of wound site between treatment group and control group (collagen I: *P* = 0.08, collagen III: *P* = 0.68) (Supplementary Fig. 7b and 7c).

#### Inflammatory markers

**Fig. 4:**
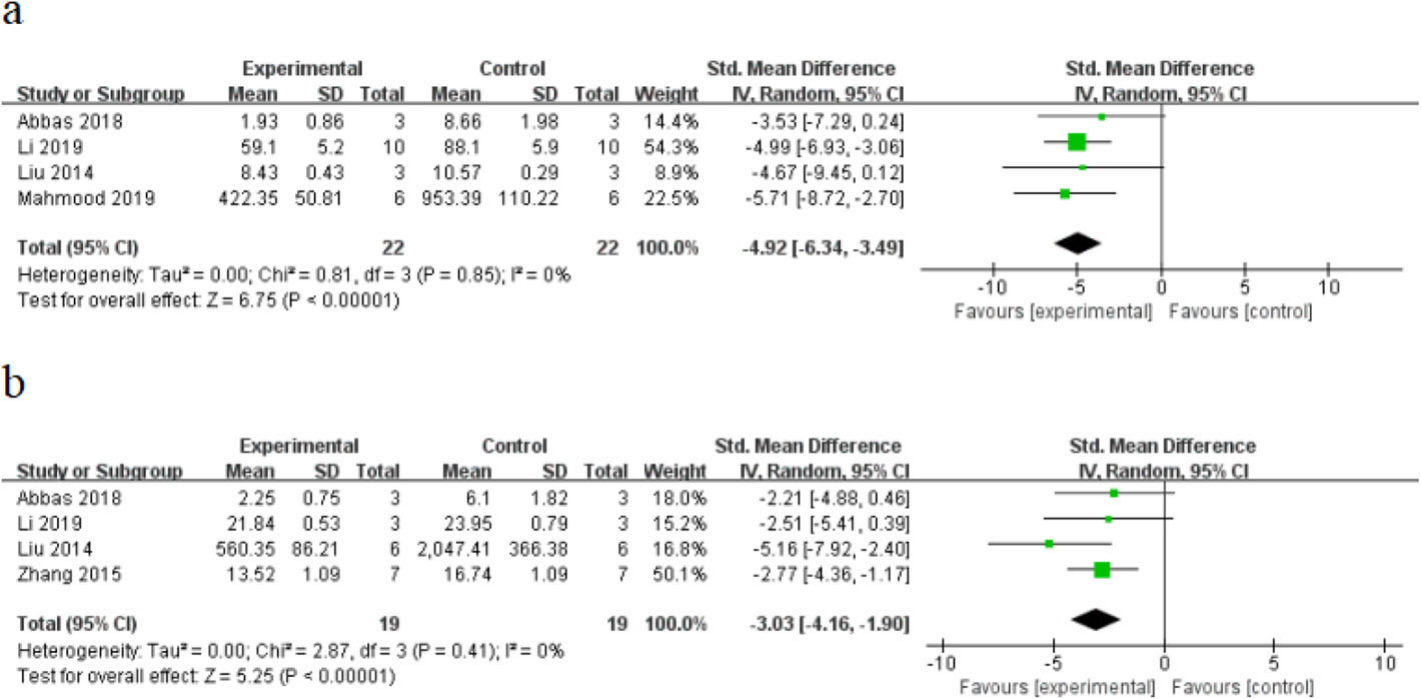
The forest plot: the effects of stem cell therapy for **a** reducing the level of IL-1 and **b** reducing the level of TNF-α of burn wounds compared with controls

Meta-analysis of four studies [29, 44, 47, 50] showed that stem cells were significant for decreasing the level of Interleukin-1 (IL-1) in burn wounds, compared with control (*n* = 44 SMD − 4.92, 95% CI (− 6.34 to − 3.49), *P* < 0.0001; *χ*^2^ = 0.81, *I*^2^ = 0%) (Fig. 4a). Meta-analysis of four studies [29, 47, 48, 50] showed that stem cells were significant for inhibiting the expression of tumor necrosis factor-α (TNF-α) in burn wounds, compared with control (*n* = 38 SMD − 3.03, 95% CI (− 4.16 to − 1.90), *P* < 0.00001; *χ*^2^ = 2.87, *I*^2^ = 0%) (Fig. 4b).

### Publication bias

**Fig. 5:**
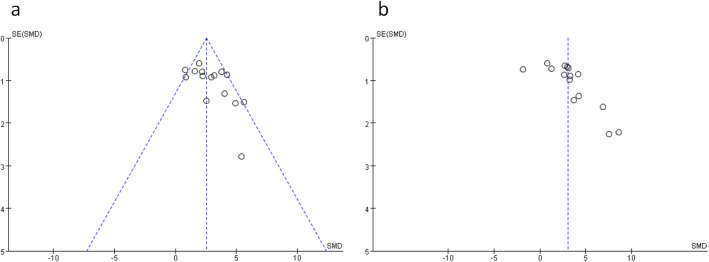
Funnel plot for **a** blood vessel density and **b** burn healing rate

Funnel plots of burn healing rate and blood vessel density were used to evaluate the publication bias. Although the funnel plot of blood vessel density was symmetrical on visual inspection (Fig. 5a), the asymmetric funnel plot of burn healing rate (Fig. 5b) showed that potential missing studies.

## Discussion

Dermal wound repair is a complex and dynamic process involving the interaction between cells and molecules, including regulation of inflammation, the formation of extracellular matrix (ECM), the release of growth factors, and angiogenesis [53]. Previous experience has shown that in order for burn wounds to heal, some of the abovementioned key steps are necessary [5]. Stem cells are known for their capacities of self-renewal and multilineage differentiation that have been regarded as a novel treatment strategy to overcome the aforementioned complications [54]. Thus, the present review aimed to provide the preclinical evidence available in the literature to elucidate the efficacy and mechanisms of stem cells for burn wounds.

To our knowledge, this is the first preclinical systematic evidences (including 21 studies and 581 animals) focused on evaluating the efficacy of stem cells for burn model animals. The present study indicated that stem cell therapy exerted the potential function of burn wound healing through anti-inflammatory action and promotion of angiogenesis. This meta-analysis article also attempted to explore heterogeneity in these included studies from different study designs of stem cell therapy, including different stem cell type, transplant type, burn degree, burn area, and treatment method in control group. Moreover, the results could be used to guide future clinical application of stem cells (e.g., cell type, transplant type).

Notably, the results showed that the main contributor to heterogeneity was burn degree on burned tissue, accounting for 54.6% of the variation. The results of subgroup analysis showed that the therapeutic effect of stem cells on the second-degree burn wound was much higher than that on the third-degree burn wound. This observation could be related to the incompletely destroyed tissue on the second-degree burn wounds, which may provide a microenvironment and nutrients for stem cells to have therapeutic effects. It is possible that in the future, stem cell therapy will be combined with other therapies that provide this environmental or nutritional benefit, which could be more conducive to the repair of severe burns.

The cell types of stem cells also contribute to partial heterogeneity. Notably, HFSCs have demonstrated better healing outcomes when treating burn wounds, compared with other cell types such as MSCs, ASCs, and ESCs. A clinical study of HFSCs applied to third-degree burns showed that HFSCs could promote dermal reepithelialization, and there were no significant differences on wound healing between dermal graft and dermoepidermal graft [55]. However, the results of meta-analysis of the treatment of ACS were considered not statistically significant (1 out of 4 reported negative effects). The small number of studies involving the use of ASCs may contribute to the result. In addition, different transplant types of stem cells showed similar efficacy. This finding maybe implicate that autologous stem cells may not be necessary for more effective treatment outcomes in animal burn treatment. None of the included studies reported rejection response due to no human lymphocyte antigen pairing. In the past few years, allogeneic stem cells have been proved to be safe and effective in many preclinical and clinical wound healing studies [56]. However, preclinical trials in the future are also required to do relevant immune experiments, which will provide more effective evidence for future clinical trials to a large extent.

We also investigated the effects of different burn area treated by stem cells, as area may have a negative effect on the healing of burn wounds. Skardal et al. [57] concluded that stem cell therapy could be an effective treatment for burns or large wounds. Patients with large-scale wounds or burns usually need more energy and nutrients to repair the wounds. In the subgroup analysis of burn area, both small (< 5 cm^2^) and large area (≥ 5 cm^2^) burns showed the effectiveness of stem cell therapy for wound healing, and the former was more obvious. Consistent with the previous research results, stem cell therapy is effective for large-scale burn wounds which means, stem cells can be used as a promising therapy in clinical large area burn patients who do not have enough skin for skin grafts.

We also explored the mechanism of stem cells in the treatment of burn wounds. Collagen deposition, as one of the key factors to determine scar hyperplasia [58], usually starts within 1 week after wound injury [59]. However, in this meta-analysis, the treatment of burn wounds with stem cells was found to have no significant effect on collagen formation. Angiogenesis is a crucial event in proper wound healing [60]. Nogami et al. [61] concluded that VEGF was activated and upregulated in the early stage of repair after skin damage and plays the role of angiogenesis. In addition, inflammatory markers such as IL-1 and TNF-α were inhibited in this meta-analysis. Although not all the mechanisms have been applied to burn wounds treated by stem cells, it is also sufficient to explain their efficacy.

Recent studies showed that the application of stem cells combined with other therapies in wound regeneration also shows positive efficacy. In particular, combined use of platelet rich plasma and SVF is reported to be effective in facial scars, chronic wounds, and soft tissue defects [62]. As reported, ASCs promote chronic wounds regeneration, possibly through promoting angiogenesis, reducing inflammation, and regulating keratinocytes to promote epithelialization [63]. Moreover, it should be noted that even with effective treatment for deep second-degree and third-degree burns, scarring is often unavoidable. Gentile et al. [64] reported autologous fat transplantation is a promising treatment for burn scars and is expected to replace traditional scar resection. In addition, numerous studies have shown that stem cells also have a good performance in other related fields, either alone or in combination with other therapies. Scioli et al. [65] found PRP will contribute to chondrogenic and osteogenic differentiation of ASCs, which may provide a new idea for the treatment of osteochondral defects in regenerative medicine. In the clinical use of HFSCs in the treatment of androgenic alopecia, the hair density of patients with androgenic alopecia increased by about 33% [66]. Gentile et al. [67] and Cervelli et al. [68] reported the application of ASCs in the treatment of soft tissue defects (ulcers and hemifacial atrophy) shows the innovative method and future prospect.

### Limitations

In our current study, some potential limitations should be mentioned when interpreting the results. First, despite our statistical analysis confirming the benefits of stem cell therapy for the healing of burn wounds, there is heterogeneity in our studies, such as stem cell type, transplantation type, and burn area. As with all meta-analyses, heterogeneity also needs to be taken into account in this study. Therefore, we conducted a subgroup analysis to determine the optimized stem cell type and the proper transplantation type, but this approach leads to a reduction in the number of studies in each subgroup. The results of meta-analysis of a small number of studies may be greatly influenced by the results of a single study. Second, our meta-analysis focused on the healing rate of burn wounds as a primary outcome. In addition to the number of vessels, secondary outcomes such as collagen deposition and inflammatory markers have been less reported. Too few studies on the same indicators may lead to the instability of the analyzed results. Because the research on stem cell therapy for burn wound is in progress, more research on these indicators may be carried out in the future. Finally, according to the qualitative assessment of funnel plot, publication bias may exist in meta-analysis. Unpublished and negative research may be the reason of publication bias.

## Conclusions

The preclinical evidences from this meta-analysis demonstrated that stem cell therapy exerts healing function for burn wounds, mainly through angiogenesis and anti-inflammatory action. We also found that there were efficacy variations, across stem cell type, burn degree, and burn area. These findings demonstrate the need for considering variations in future clinical studies using stem cells to treat a burn wound in order to maximize the effectiveness. In general, stem cells can potentially become a novel therapy candidate for burn wounds.

## Supplementary information

**Additional file 1.** PRISMA 2009 checklist

**Additional file 2. Supplementary Figure 1.** Subgroup analyses of cell type regarding stem cell therapy in animal model of burn wounds for the primary outcome of healing rate.

**Additional file 3. Supplementary Figure 2.** Subgroup analyses of transplant type regarding stem cell therapy in animal model of burn wounds for the primary outcome of healing rate.

**Additional file 4. Supplementary Figure 3.** Subgroup analyses of burn degree regarding stem cell therapy in animal model of burn wounds for the primary outcome of healing rate.

**Additional file 5. Supplementary Figure 4.** Subgroup analyses of burn area regarding stem cell therapy in animal model of burn wounds for the primary outcome of healing rate.

**Additional file 6. Supplementary Figure 5.** Subgroup analyses of treatment methods in the control group regarding stem cell therapy in animal model of burn wounds for the primary outcome of healing rate.

**Additional file 7. Supplementary Figure 6.** Subgroup analyses of species regarding stem cell therapy in animal model of burn wounds for the primary outcome of healing rate.

**Additional file 8. Supplementary Figure 7.** The forest Plot: the effects of stem cell therapy for (a) total collagen deposition, collagen (b) I and (c) III deposition on burn wounds compared with controls.

## Data Availability

The data supporting the conclusions of this article are all online.

## Abbreviations

ASCs: Adipose-derived stem cells
CI: Confidence Interval
ECM: Extracellular matrix
ESCs: Epidermal stem cells
HFSCs: Hair follicle stem cells
IL-1: Interleukin-1
LR: Lactate ringer
MSCs: Mesenchymal stem cells
NA: Not available
PBS: Phosphate-buffered saline
RCT: Randomized controlled trial
SD: Sprague Dawley
SMD: Standard mean difference
SVF: Stromal vascular fraction
TNF-α: Tumor necrosis factor-α
VEGF: Vascular endothelial growth factor
