# Supplementary figures and images for: Efficacy of stem cell therapy for burn wounds: a systematic review and meta-analysis of preclinical studies
Source: Stem Cell Res Ther. 2020 Jul 29;11:322. doi: 10.1186/s13287-020-01839-9 (PMC7389817; doi:10.1186/s13287-020-01839-9)

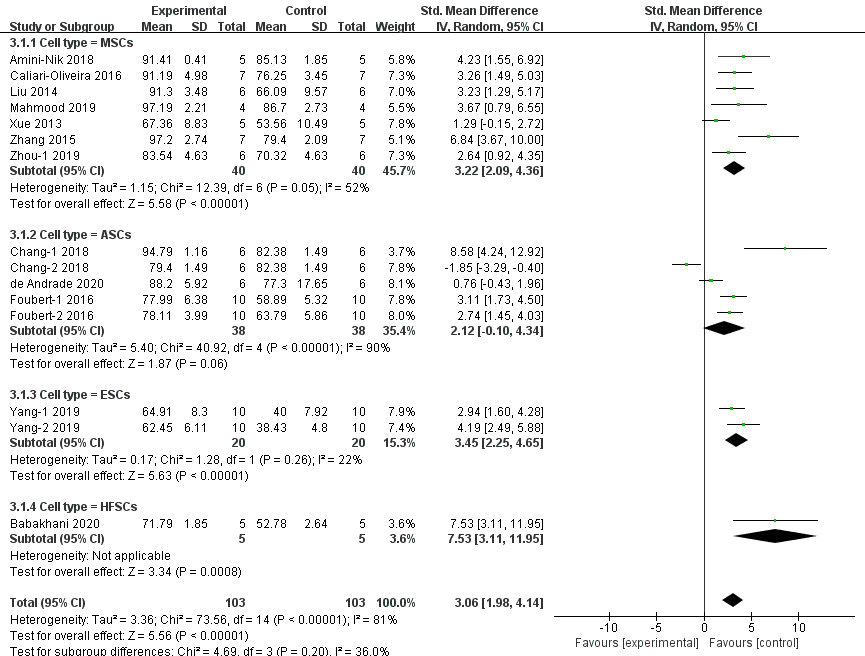

Supplement: Supplementary file 2 — Additional file 2. Supplementary Figure 1. Subgroup analyses of cell type regarding stem cell therapy in animal model of burn wounds for the primary outcome of healing rate. [file 13287_2020_1839_MOESM2_ESM.png]

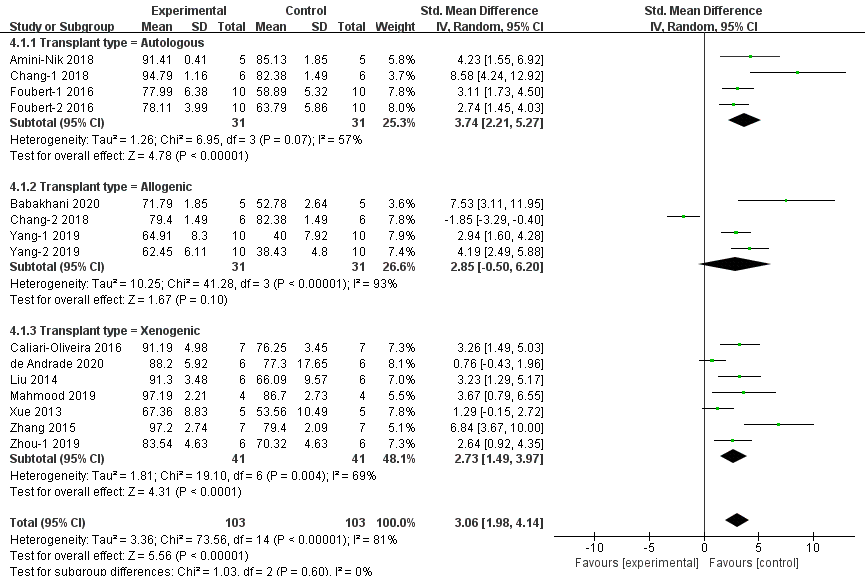

Supplement: Supplementary file 3 — Additional file 3. Supplementary Figure 2. Subgroup analyses of transplant type regarding stem cell therapy in animal model of burn wounds for the primary outcome of healing rate. [file 13287_2020_1839_MOESM3_ESM.png]

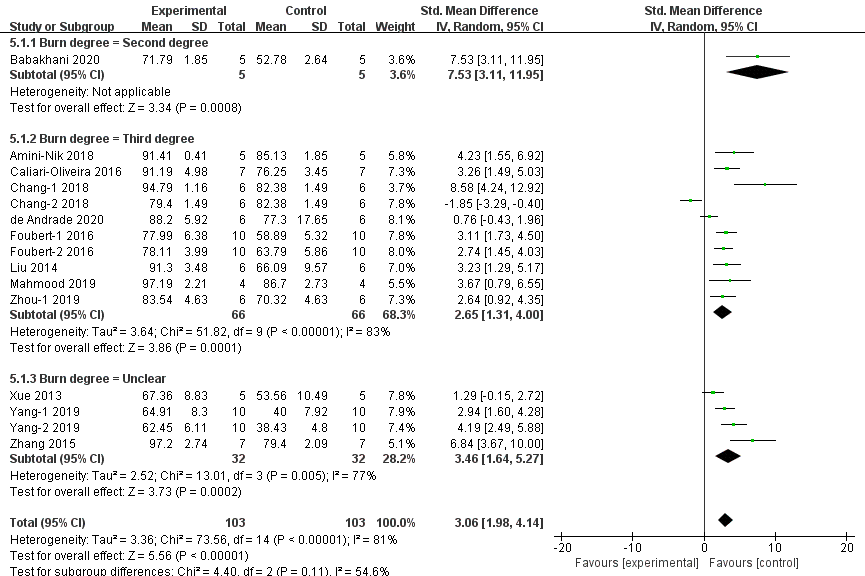

Supplement: Supplementary file 4 — Additional file 4. Supplementary Figure 3. Subgroup analyses of burn degree regarding stem cell therapy in animal model of burn wounds for the primary outcome of healing rate. [file 13287_2020_1839_MOESM4_ESM.png]

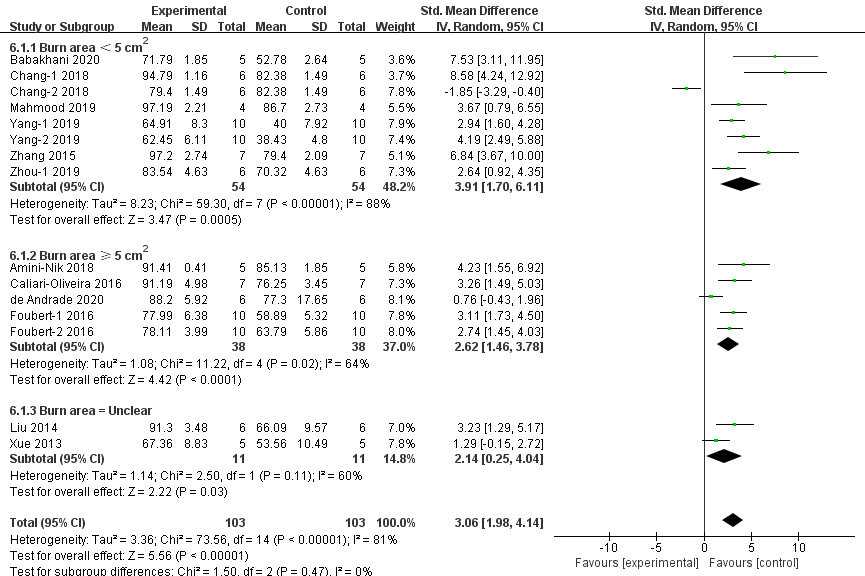

Supplement: Supplementary file 5 — Additional file 5. Supplementary Figure 4. Subgroup analyses of burn area regarding stem cell therapy in animal model of burn wounds for the primary outcome of healing rate. [file 13287_2020_1839_MOESM5_ESM.png]

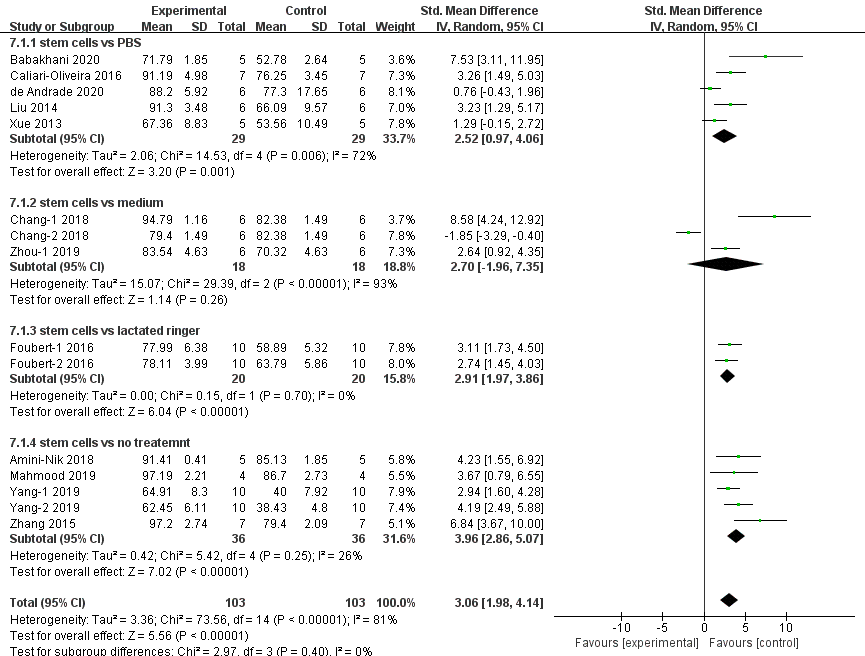

Supplement: Supplementary file 6 — Additional file 6. Supplementary Figure 5. Subgroup analyses of treatment methods in the control group regarding stem cell therapy in animal model of burn wounds for the primary outcome of healing rate. [file 13287_2020_1839_MOESM6_ESM.png]

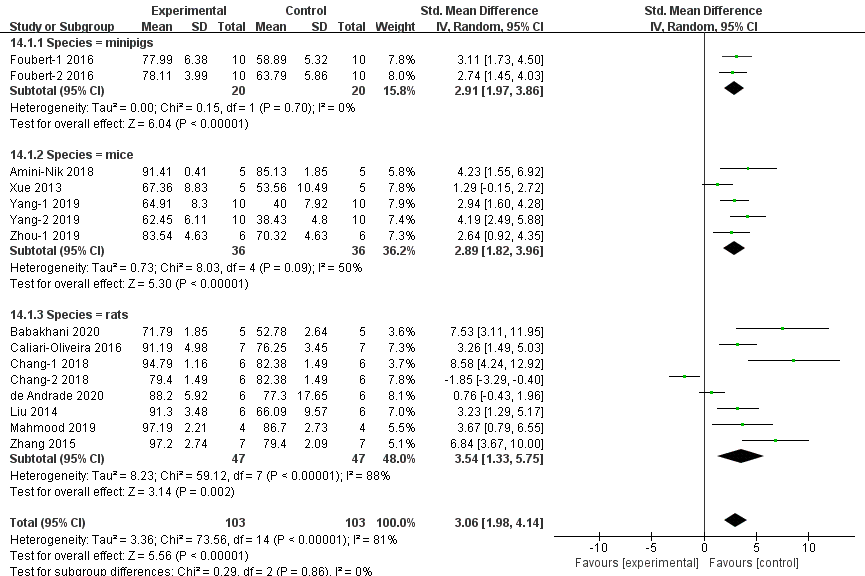

Supplement: Supplementary file 7 — Additional file 7. Supplementary Figure 6. Subgroup analyses of species regarding stem cell therapy in animal model of burn wounds for the primary outcome of healing rate. [file 13287_2020_1839_MOESM7_ESM.png]

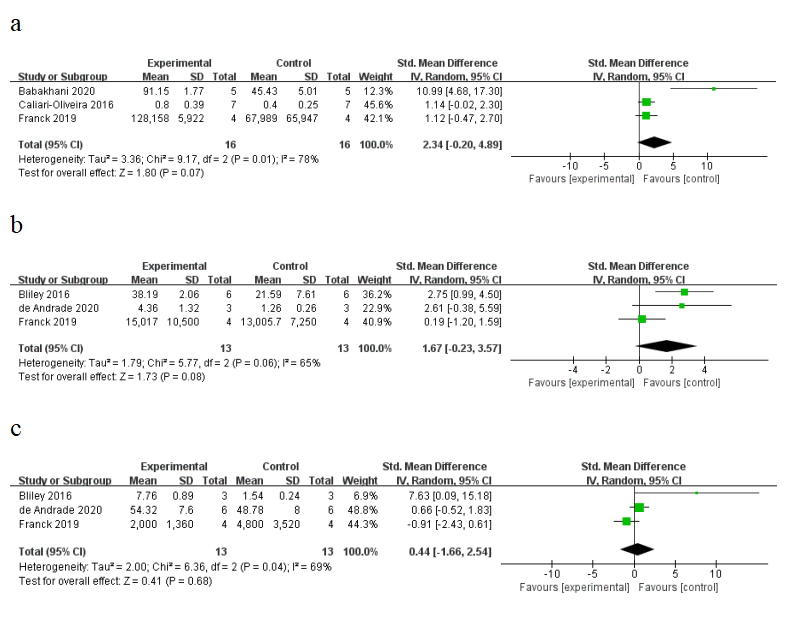

Supplement: Supplementary file 8 — Additional file 8. Supplementary Figure 7. The forest Plot: the effects of stem cell therapy for (a) total collagen deposition, collagen (b) I and (c) III deposition on burn wounds compared with controls. [file 13287_2020_1839_MOESM8_ESM.png]
